# Pre-exposure prophylaxis among Brazilian men who have sex with men: a comparative study between migrants and non-migrants

**DOI:** 10.3389/fpubh.2023.1198339

**Published:** 2023-08-17

**Authors:** Álvaro Francisco Lopes Sousa, Shirley Verônica Melo Almeida Lima, Caíque Jordan Nunes Ribeiro, Anderson Reis de Sousa, Emerson Lucas Silva Camargo, Layze Braz de Oliveira, João Cruz Neto, Inês Fronteira, Isabel Amélia Costa Mendes

**Affiliations:** ^1^Hospital Sírio-Libânes, Instituto de Ensino e Pesquisa, São Paulo, Brazil; ^2^Global Health and Tropical Medicine, Instituto de Higiene e Medicina Tropical, Universidade Nova de Lisboa, Lisbon, Portugal; ^3^Collective Health Research Center, Universidade Federal de Sergipe, Lagarto, Sergipe, Brazil; ^4^Nursing Graduate Program, Universidade Federal de Sergipe, São Cristóvão, Sergipe, Brazil; ^5^Graduate Program in Nursing and Health of the Nursing School, Universidade Federal da Bahia, Salvador, Bahia, Brazil; ^6^Ribeirão Preto College of Nursing, Universidade de São Paulo, Ribeirão Preto, São Paulo, Brazil; ^7^University for International Integration of the Afro-Brazilian Lusophony, Redenção, Ceará, Brazil; ^8^Public Health Research Centre, Comprehensive Health Research Center, National School of Public Health, New University of Lisbon, Lisbon, Portugal

**Keywords:** PrEP, HIV, MSM, sexual behavior, migrant populations, international migration, global health

## Abstract

**Introduction:**

Investigating the use and adherence to pre-exposure prophylaxis (PrEP) in MSM is a global health priority in the fight against HIV. Strategies must be capable of increasing usage and reaching not only the population living in the country but also those who immigrate, who face additional vulnerabilities. Based on this, in this observational, cross-sectional, and analytical study, our aim is to analyze the use of PrEP among Brazilian men who have sex with men, whether they are migrants or not. We aim to highlight preventive opportunities and strategies for the global health scenario.

**Methods:**

We utilized a sample of Brazilians living in the country and Brazilian immigrants residing in Portugal, one of the main destinations for Brazilians in recent years. To estimate the prevalence ratio (PR) of PrEP use, we employed the Poisson regression model with robust variance estimation using a covariance matrix.

**Results:**

A total of 1,117 Brazilian MSM PrEP users participated in this study, with 788 residing in Brazil and 328 in Portugal. Multivariate analysis was conducted in three stages: overall, and for subgroups of residents in Brazil and immigrants in Portugal. We identified four convergent factors that increased the prevalence of PrEP use in Brazilians regardless of migration status: having two or more casual sexual partners per month, engaging in challenging sexual practices as the receptive partner, disclosing serological status on apps, and being single. Among native Brazilians, four unique factors stood out: being in a polyamorous relationship, having sexual relations with unknown casual partners, and having higher levels of education.

**Discussion:**

This study highlights the need to implement strategies to strengthen PrEP adherence in Brazil and create international programs that facilitate its usage among populations migrating between these two countries.

## 1. Introduction

Human immunodeficiency virus (HIV) infection remains a challenge for healthcare systems, and while the global implementation of HIV treatment has saved millions of lives, efforts to prevent new HIV infections have been less successful. According to UNAIDS, the annual number of new HIV infections among adults worldwide has remained virtually unchanged over the past 4 years, and the total number of new infections has only decreased by 31% since 2010, well below the United Nations General Assembly’s target of a 75% reduction by the year 2020 (1).

Latin America has a particular case, with an estimated 2.2 million [Confidence Interval—CI: 1.5–2.8 million] people living with HIV/AIDS, and the region has not seen any reduction in infections over the last decade. Brazil plays a key role in this scenario, because it accounts for 35% of the total population of the region and concentrates 47% of new infections. Since the establishment of compulsory reporting of HIV cases until the year 2021, Brazil has reported 381,793 cases, with an annual average increase of 36.8 thousand new cases of HIV/AIDS in the last 5 years (2).

Brazil is the Latin American country that invests the most in prevention, with efforts mainly focused on key populations, such as men who have sex with men (MSM), sex workers, and transgender people. However, financial investment in the pillars of primary prevention remains insufficient, and pre-exposure prophylaxis (PrEP), although available through the public sector, is underutilized in the country (3).

The implementation of PrEP in the Brazilian Unified Health System (SUS) began in December 2017 and gradually expanded throughout the country, especially in the first 3 years of the policy. From January 2018 to December 2020, 158,836 PrEP dispensations were made, with 16,938 continuous PrEP users since then (4). Although official reports provide a good characterization of PrEP dispensations, few data focus on the reasons why individuals continue to use PrEP or the factors determining the suspension of its use.

In Brazil, PrEP is recommended for HIV-negative people who are at high risk of HIV infection. This includes people who do not regularly use condoms during sexual contact, whether anal or vaginal, people who have used post-exposure prophylaxis (PEP) for HIV repeatedly, people with recurrent sexually transmitted infections (STIs), or those engaged in chemsex practices, for example (5). PrEP is only provided free of charge at local specialty health services after regular health surveillance, HIV testing, and other screenings for other STDs, all done by a qualified healthcare professional (5). Although the target audience is the populations most vulnerable to HIV infection, notably MSM, transgender people, and sex workers, as well as people with HIV-discordant partnerships, some other key populations, such as immigrants, do not have priority in the Brazilian PrEP policy (6).

The commercial, social, and population relations between Brazil and Portugal are centuries-old. Both are members of the Community of Portuguese-Speaking Countries and receive the most immigrants within this community. Despite Portugal having one of the most comprehensive legal frameworks for protecting immigrants in the world, including health protection and social security, there are difficulties in enforcing political, economic, and even social rights that align with current legal systems (7, 8). This also applies to public health and strategies for HIV/AIDS prevention and control.

In Portugal, regardless of immigrant status (legal or illegal), healthcare related to HIV treatment is free and partly available in both the National Health Service (SNS) and Non-Governmental Organizations, subject, however, to a residency period of more than 90 days in the country and the presentation of an Address Certificate issued by the Parish Council of their area of residence (9). In Brazil, immigrants have access to free PrEP, provided they are referred to an HIV consultation at a health service, which is not always an easy task (6).

Nevertheless, studies conducted with MSM have revealed difficulties in accessing PrEP in Portugal, particularly regarding the ideal way to enter the system, the waiting time for the first consultation, or even approval for treatment. This has led, for example, to the purchase of these drugs online at high prices and their use without proper monitoring, something that may not be possible for immigrants (10–12).

Despite all the social, political, and cultural differences, Brazil and Portugal have a similar policy for preventing HIV/AIDS, especially regarding PrEP, except for the already clarified differences. However, there is no mechanism that facilitates the use of PrEP by immigrants in these countries, such as a policy for continued use by immigrants upon entering a new country or a specific process to determine the requirements for PrEP use by immigrants. This constitutes an important programmatic vulnerability considering that the immigrant population is growing in both countries and is doubly invisible regarding HIV/AIDS.

In this regard, this research aims to focus on the factors that contribute to PrEP adherence among Brazilians and to understand if these factors change in the context of immigration to another country. By doing so, it aims to provide evidence for the proposition of global public policies that enable the continued use of PrEP among immigrants. Therefore, this study analyzes the use of PrEP by Brazilian men who have sex with men, whether migrants or not, highlighting preventive opportunities and strategies for the global health scenario.

## 2. Methods

### 2.1. Type of study

This is an observational, cross-sectional, and analytical study, a section of the “In_PrEP” project: an international research project conducted throughout Brazil and Portugal from January 2020 to May 2021, led by the Institute of Hygiene and Tropical Medicine (IHMT) in partnership with the University of São Paulo (USP), some of whose results have already been published (6).

### 2.2. Population, sample, and eligibility criteria

The study included of Brazilian MSM over 18 years of age, who have resided in Brazil and Portugal for at least 3 months. A sample size calculation was performed using G*Power software (version 3.1.9.7) to determine the required sample size for the study. The calculation considered the population of brazilian, assuming a presumed prevalence of the outcome of 50% (aiming to maximize the sample size, considering the lack of prevalence data) (13, 14), a tolerable standard error of 3%, and a confidence level of 95%.

### 2.3. Data collection procedures

Participants were recruited online using a “snowball” sampling adapted to the virtual environment and consolidated by other studies (15–19). Through this method, participants themselves are responsible for recruiting other individuals with similar situations through their social networks and contacts. Following the method’s criteria, we selected 30 MSM with different characteristics, namely: region or district of residence, skin color (white/non-white), income, and educational level (elementary, high school, or university) (16, 19). These were the first participants, called “seeds.”

Each participant received a link to the survey and was instructed to invite other MSM from their social network. The seeds were identified through two location-based dating apps (Grindr and Hornet) via direct chat with online users. The first available individuals online on each of the two apps who met the inclusion criteria were included, as recommended by previous studies (15, 16, 20).

At the same time, the researchers also promoted the study on two social networks, Facebook and Instagram, targeting MSM over 18 years of age in both countries. Social networks were used as an additional resource due to their ability to access people located in remote areas, which is absolutely necessary in the case of a continental country like Brazil. Only individuals who identified themselves as men (cisgender or transgender) and aged 18+ were included. Tourists and non-Portuguese speakers were excluded.

### 2.4. Data collection instrument

The survey form was hosted on the Surveymonkey platform for data collection in two versions, which has security features that allow only one response per Internet Protocol (IP). As there are significant linguistic differences between the two countries studied, the form was made available in two versions: Brazilian Portuguese and European Portuguese. Prior to this, the form was validated (face-content validation) by 10 expert judges in the field, five from each country. There was also a pre-test with five participants from each country.

The form was divided into five sections with 40 questions, mostly multiple-choice, with some mandatory questions to proceed. The questions covered social and demographic information (gender identity, sexual orientation, age, education level, country of residence, country of origin, and length of residence in the country), sexual and affective relationships (type of partners, type of relationship, and number of partners), knowledge about HIV/AIDS prevention methods, sexual behavior and practices, protective measures adopted, search and use of health services, consumption, and willingness to use PrEP (at time of research).

### 2.5. Variables and measures

The outcome of this study was the use of PrEP among MSM (Yes/No) self-reported by the participants which was examined with the following question: “Have you taken, as prescribed by the health professional, a daily PrEP tablet in the last 30 days?” For this definition, we followed the official recommendations regarding adherence: “the ideal use of antiretroviral drugs in the closest possible way to that prescribed by the health team, respecting the doses and times” (6).

To comprehend the factors associated with PrEP use, an investigation was conducted on social and demographic characteristics, as well as variables pertaining to sexual and affective relationships, HIV testing and status, serophobia (fear of disclosing one’s HIV serological status), recent sexual behaviors and practices (within the last 30 and 60 days), persistence in condom use (defined as use during the most recent sexual intercourse), and reasons for not using condoms.

The following practices were defined based on previous studies (6, 7, 19):

### 2.6. Data analysis

The data were analyzed using the Statistical Package for the Social Sciences (SPSS) version 24.0 (SPSS Inc., Chicago, IL, United States).

Initially, we conducted an exploratory analysis using descriptive statistics and the Pearson chi-square test to verify the association between the predictor variables and the country of residence of the study participants (Brazil vs. Portugal). The data were represented using absolute frequencies, and the percentage frequencies were calculated considering the total value established in the columns as the denominator (Total Brazilian MSM = 5,467; Brazilian MSM living in Brazil = 4,434; Brazilian MSM living in Portugal = 1,033).

Subsequently, a bivariate analysis was performed, employing Pearson’s chi-square test to select variables eligible for inclusion in the multivariate model, considering a value of *p* < 0.20, theoretical relevance, or better adjustment conditions in the final model. Additionally, we calculated crude prevalence ratios (PRs) with their corresponding 95% confidence intervals (CIs) to measure the strength and direction of the association between the outcome and predictor variables. PR was chosen as the measure of association since the frequency of the outcome of interest was >10%. Thus, estimating odds ratios (ORs) through logistic regression modeling could overestimate the strength of associations between the outcome and independent variables.

To identify factors independently associated with PrEP use, we employed a multivariate generalized linear regression model using Poisson regression with a log-linear link function, hybrid parameter estimation method, robust variance estimator, and Type III analysis for model testing. The adherence to the Poisson distribution was tested using the Kolmogorov–Smirnov test (value of *p* > 0.05), and the assumption of equidispersion was met as the variance and mean of the outcome had similar values.

The omnibus test was used to assess the overall significance of the multivariate model compared to a model consisting solely of the intercept (value of *p* < 0.05). The Akaike Information Criterion (AIC), deviance, and log-likelihood were employed as reference criteria for selecting the best-fitting model, with lower values indicating better fit. The significance of estimates for variables included in the final model was examined using the Wald chi-square test. Adjusted prevalence ratios (aPRs) and their corresponding 95% CIs were calculated. Variables with a value of *p* < 0.05 in the final model were considered statistically significant. All variables were analyzed to assess multicollinearity, following tolerance coefficients and VIF (variance inflation factor) parameters (tolerance > 0.01; VIF < 4).

### 2.7. Ethical considerations

The research was approved by the Research Ethics Committee of the IHMT of Universidade Nova de Lisboa (Protocol n° 12.19/2020), as well as by the Research Ethics Committee of the School of Nursing of Ribeirão Preto, University of São Paulo, Brazil (Protocol No. 4,163,084). For the development of this study, all ethical norms in force in both countries were respected through the online application of a Free and Informed Consent Form to obtain the consent of the participants. At the end of the survey, we provided access to institutional sites to obtain various information on HIV/AIDS prevention.

## 3. Results

5,467 Brazilian MSM participated in this study, of whom 4,434 (81.12%) were Brazilian residents and 1,033 (18.9%) were immigrants living in Portugal. In this sample, 1,116 Brazilians were using PrEP, with a prevalence of 20.4%, of which 788 (17.8%) were Brazilian residents in Brazil and 328 (31.7%) were Brazilian residents in Portugal. Table 1 presents the characterization of the study participants according to their country of residence. The sample was predominantly composed of young adult MSM (4,457; 81.5%), with a higher education level (4,126; 75.5%), homosexual orientation (4,472; 81.8%), and having a steady partner (3,779; 69.1%). Out of the four social and demographic characteristics analyzed, only age was found to be associated with the participants’ country of residence, as demonstrated in Table 1.

**Table 1:** Baseline characteristics of Brazilian MSM.

Variables		Brazilian MSM						*p* value
		Living in Brazil (*n* = 4,434)		Living in Portugal (*n* = 1,033)		Total (*n* = 5,467)		
		*n*	%	*n*	%	*n*	%	
Social and demographic characteristics								
Age	<35 years	3,532	79.7	925	89.5	4,457	81.5	<0.001
	≥35 years	902	20.3	108	10.5	1,010	18.5	
Education	Low education	1,090	24.6	251	24.3	1,341	24.5	0.848
	Higher education	3,344	75.4	782	75.7	4,126	75.5	
Bisexual	Yes	811	18.3	184	17.8	995	18.2	0.720
	No	3,623	81.7	849	82.2	4,472	81.8	
Type of relationship	Single	1,114	25.1	278	26.9	1,392	25.5	0.145
	Polyamorous relationship	231	5.2	65	6.3	296	5.4	
	Steady partner	3,089	69.7	690	66.8	3,779	69.1	
Sexual partnerships								
Declared serological status on mobile apps	Yes	882	19.9	247	23.9	1,129	20.7	0.005
	No	3,552	80.1	786	76.1	4,338	79.3	
Number of sexual partners in the last 30 days	None	418	9.4	94	9.1	512	9.4	<0.001
	1	1,678	37.8	160	15.5	1,838	33.6	
	≥2	2,338	52.7	779	75.4	3,117	57.0	
Recent HIV testing (last 12 months)	Yes	2,995	67.5	699	67.7	3,694	67.6	0.970
	No	1,439	32.5	334	32.3	1,773	32.4	
Consistent use of condoms	Yes	416	9.4	84	8.1	500	9.1	0.232
	No	4,018	90.6	949	91.9	4,967	90.9	
Had a HIV+ partner (last 12 months)	Yes	88	2.0	16	1.5	104	1.9	0.426
	No	4,346	98.0	1,017	98.5	5,363	98.1	
Reasons for not using condoms								
Partner uses PrEP	Yes	382	8.6	130	12.6	512	9.4	<0.001
	No	4,052	91.4	903	87.4	4,955	90.6	
Unknown partner	Yes	1,262	28.5	264	25.6	1,526	27.9	0.066
	No	3,172	71.5	769	74.4	3,941	72.1	
Partner reports no STI	Yes	443	10.0	104	10.1	547	10.0	0.987
	No	3,991	90.0	929	89.9	4,920	90.0	
Partner reports recent HIV testing	Yes	4,031	90.9	962	93.1	4,993	91.3	0.027
	No	403	9.1	71	6.9	474	8.7	
Sexual practices
Group sex	Yes	1,835	41.4	413	40.0	2,248	41.1	0.429
	No	2,599	58.6	620	60.0	3,219	58.9	
Frequent bareback	Yes	2,566	57.9	653	63.2	3,219	58.9	0.002
	No	1,868	42.1	380	36.8	2,248	41.1	
Chemsex	Yes	1,264	28.5	328	31.8	1,592	29.1	0.042
	No	3,170	71.5	705	68.2	3,875	70.9	
Fisting/footing	Yes	381	8.6	104	10.1	485	8.9	0.150
	No	4,053	91.4	929	89.9	4,982	91.1	
Cruising	Yes	241	5.4	162	15.7	403	7.4	<0.001
	No	4,193	94.6	871	84.3	5,064	92.6	
Double penetration	Yes	942	21.2	297	28.8	1,239	22.7	<0.001
	No	3,492	78.8	736	71.2	4,228	77.3	

Table 2 presents bivariate analyses of the characteristics of Brazilian MSM and their respective sub-samples (residents in Brazil and Brazilian immigrants living in Portugal). Considering the overall sample and PrEP use, it was identified that out of the 23 sociodemographic and sexual behavior characteristics, 17 variables were associated with a higher prevalence of PrEP use. It is noteworthy that participants who were single (PR: 1.87) and those who engaged in polyamorous relationships (PR: 3.63) were associated with a higher prevalence of PrEP use. Individuals who disclose their HIV status on mobile apps have a 5.18 times higher prevalence of PrEP use. Those who reported group sex had a 27% lower prevalence of PrEP use.

**Table 2:** Bivariate analysis of factors associated with the use of PrEP among men who have sex with men (MSM), 2021.

Variables		PrEP use					
		Brazilian MSM (*n* = 1,116)		Brazilian MSM living in Brazil (*n* = 788)		Brazilian MSM living in Portugal (*n* = 328)	
		*n* (%)	PR (CI95%)	*n* (%)	PR (CI95%)	*n* (%)	PR (CI95%)
			*p* value		*p* value		*p* value
Social and demographic characteristics							
Age	< 35 years	905 (20.3)	1.03 (0.90–1.18)	632 (17.9)	1.03 (0.88–1.21)	273 (29.5)	0.58 (0.47–0.71)
	≥35 years^[ref]^	211 (20.9)	0.677	156 (17.3)	0.675	55 (50.9)	<0.001
Education	Low education	307 (22.9)	1.17 (1.04–1.31)	243 (22.3)	1.36 (1.19–1.56)	64 (25.5)	0.75 (0.60–0.95)
	Higher education^[ref]^	809 (19.6)	0.009	545 (16.3)	<0.001	264 (33.8)	0.018
Bissexual	Yes	230 (23.1)	1.17 (1.03–1.33)	172 (21.2)	1.24 (1.07–1.45)	58 (31.5)	0.99 (0.78–1.25)
	No^[ref]^	886 (19.8)	0.019	616 (17.0)	0.004	270 (31.8)	0.941
Type of relationship	Single	827 (21.9)	1.87 (1.60–2.18)	590 (19.1)	2.01 (1.65–2.44)	237 (34.3)	1.67 (1.30–2.16)
			<0.001		<0.001		<0.001
	Polyamorous relationship	126 (42.6)	3.63 (2.99–4.42)	92 (39.8)	4.19 (3.29–5.32)	34 (52.3)	2.55 (1.84–3.54)
			<0.001		<0.001		<0.001
	Steady partner ^[ref]^	163 (11.7)	-	106 (9.5)	-	57 (20.5)	-
Sexual partnerships							
Declared serological status on mobile apps	Yes	641 (56.8)	5.18 (4.69–5.72)	444 (50.3)	5.20 (4.61–5.86)	197 (79.8)	4.78 (4.04–5.66)
	No^[ref]^	475 (10.9)	<0.001	344 (9.7)	<0.001	131 (16.7)	<0.001
Number of sexual partners in the last 30 days	None^[ref]^	6 (1.2)	-	3 (0.7)	-	3 (3.2)	-
	1	16 (0.9)	0.74 (0.29–1.89)	12 (0.7)	1.0 (0.28–3.51)	4 (2.5)	0.78 (0.17–3.42)
			0.532		0.995		0.745
	≥2	1,094 (35.1)	29.95 (13.50–66.45)	773 (33.1)	46.1 (14.89–142.46)	321 (41.2)	12.9 (4.22–39.43)
			<0.001		<0.001		<0.001
Recent HIV testing (last 12 months)	Yes	1,102 (29.8)	37.78 (22.37–63.80) <0.001	783 (26.1)	75.24 (31.30–180.86)	319 (45.6)	16.94 (8.85–32.43)
	No^[ref]^	14 (0.8)		5 (0.3)	<0.001	9 (2.7)	<0.001
Consistent use of condoms	Yes	116 (23.2)	1.15 (0.97–1.36)	84 (20.2)	1.15 (0.94–1.41)	32 (38.1)	1.22 (0.91–1.63)
	No^[ref]^	1,000 (20.1)	0.116	704 (17.5)	0.170	296 (31.2)	0.174
Had a HIV+ partner (last 12 months)	Yes	23 (22.1)	1.09 (0.75–1.56)	19 (21.6)	1.22 (0.81–1.82)	4 (25.0)	0.78 (0.33–1.84)
	No^[ref]^	1,093 (20.4)	0.625	769 (17.7)	0.333	324 (31.9)	0.580
Reasons for not using condoms							
Partner uses PrEP	Yes	177 (34.6)	1.82 (1.60–2.08)	122 (31.9)	1.94 (1.65–2.28)	55 (42.3)	1.40 (1.11–1.75)
	No^[ref]^	939 (19.0)	<0.001	666 (16.4)	<0.001	273 (30.2)	0.003
Unknown partner	Yes	850 (21.6)	1.23 (1.10–1.40)	592 (18.7)	1.20 (1.04–1.39)	258 (33.6)	1.26 (1.01–1.58)
	No^[ref]^	266 (17.4)	0.001	196 (15.5)	0.015	70 (26.5)	0.040
Partner reports no STI	Yes	114 (20.8)	1.02 (0.86–1.21)	84 (19.0)	1.07 (0.87–1.31)	30 (28.8)	0.89 (0.65–1.23)
	No^[ref]^	1,002 (20.4)	0.780	704 (17.6)	0.487	298 (32.1)	0.510
Partner reports recent HIV testing	Yes^[ref]^	1,037 (20.8)	0.80 (0.65–0.99)	725 (18.0)	0.87 (0.68–1.10)	312 (32.4)	0.69 (0.45–1.08)
	No	79 (16.7)	0.039	63 (15.6)	0.245	16 (22.5)	0.105
Sexual practices							
Group sex	Yes	375 (16.7)	0.73 (0.65–0.81)	261 (14.2)	0.70 (0.61–0.80)	114 (27.6)	0.80 (0.66–0.96)
	No^[ref]^	741 (23.0)	<0.001	527 (20.3)	<0.001	214 (34.5)	0.021
Frequent bareback	Yes	792 (24.6)	1.70 (1.52–1.92)	551 (21.5)	1.69 (1.47–1.95)	241 (36.9)	1.61 (1.30–1.99)
	No^[ref]^	324 (14.4)	<0.001	237 (12.7)	<0.001	87 (22.9)	<0.001
Chemsex	Yes	428 (26.9)	1.51 (1.36–1.68)	288 (22.8)	1.44 (1.27–1.64)	140 (42.7)	1.60 (1.34–190)
	No^[ref]^	688 (17.8)	<0.001	500 (15.8)	<0.001	188 (26.7)	<0.001
Fisting/footing	Yes	127 (26.2)	1.32 (1.13–1.55)	86 (22.6)	1.30 (1.07–1.59)	41 (39.4)	1.27 (0.98–1.65)
	No^[ref]^	989 (19.9)	0.001	702 (17.3)	0.009	287 (30.9)	0.063
Cruising	Yes	304 (75.4)	4.70 (4.32–5.12)	186 (77.2)	5.38 (4.86–5.94)	118 (72.8)	3.02 (2.60–3.51)
	No^[ref]^	812 (16.0)	<0.001	602 (14.4)	<0.001	210 (24.1)	<0.001
Double penetration	Yes	311 (25.1)	1.32 (1.20–1.48)	196 (20.8)	1.23 (1.06–1.41)	115 (38.7)	1.34 (1.11–1.60)
	No^[ref]^	805 (19.0)	<0.001	592 (17.0)	0.005	213 (28.9)	0.002

[ref] Category of reference.

In the bivariate analysis corresponding to Brazilians living in Brazil, it was noticeable that sexual behaviors such as barebacking (RP: 1.69), chemsex (RP: 1.44), cruising (RP: 5.38), fisting (RP: 1.30), and double penetration (RP: 1.23) were more prevalent for PrEP use, characterizing that individuals who engage in challenging sexual practices tend to use PrEP more. Participants with low education, those bissexual, those unaware of their serostatus, and those with relationships with unknown partners were also associated with higher PrEP use among Brazilians living in Brazil (Table 1).

Brazilian immigrants living in Portugal presented distinct characteristics regarding higher PrEP use prevalence compared to Brazilian residents. It is noteworthy that younger individuals and those with low education had 42 and 25% lower prevalence of using PrEP, respectively, compared to immigrants who were older and had higher levels of education. Disclosing serostatus on mobile apps and having more than two sexual partners in the last 30 days were associated with approximately 5- and 13-times higher PrEP use frequency, respectively. Similarly, to Brazilians living in Brazil, immigrants practicing chemsex, fisting, cruising, and double penetration also showed higher PrEP use (Table 2).

Multivariate analysis was conducted in three stages. In the first stage, a modeling was performed for the general sample, and in the second and third stages, we executed an analysis with the subgroups of residents in Brazil and immigrants living in Portugal in order to verify the existence of similarities or differences between the two models developed. In the general model, out of the 17 eligible variables, 13 were selected for inclusion in the final model. For the subgroup of residents in Brazil, 14 were eligible and nine were selected, while for the immigrant subgroup, 13 were eligible and five were selected. Variables were selected based on criteria of best model performance. Routine HIV testing variables as well as having known HIV status were adjusted for better performance of the statistical model.

Considering all Brazilian participants, a higher prevalence of PrEP use was associated with having more than two sexual partners in 30 days (aPR: 19.40), declaring serological status on dating apps (aPR: 2.21), being in a polyamorous relationship (aPR: 1.61), being single (aPR: 1.32), being bisexual (aPR: 1.33), engaging in challenging sexual practices (aPR: 1.26), having sex with an unknown partner (aPR: 1.21), and practicing barebacking recurrently (aPR: 1.13; Table 3).

**Table 3:** Multivariate analysis of factors associated with the use of PrEP among MSM from Brazil and Portugal, 2021.

Variables	β	aPR	CI95%		*p* value
			Lower	Superior	
*General multivariate analysis* ^1^					
≥2 casual sexual partners per month	2.965	19.40	8.71	43.20	<0.001
Disclosing serological status on dating apps	0.791	2.21	2.01	2.41	<0.001
Being in a polyamorous relationship	0.477	1.61	1.40	1.86	<0.001
Being bisexual	0.288	1.33	1.22	1.46	<0.001
Being single	0.274	1.32	1.16	1.48	<0.001
*Engaging in challenging sexual practices	0.233	1.26	1.16	1.37	<0.001
Having sex with unknown partners	0.194	1.21	1.10	1.33	<0.001
Engaging in recurrent bareback sex	0.123	1.13	1.02	1.25	0.015
Group sex	−0.112	0.90	0.81	0.98	0.020
*Brazilian residents multivariate analysis* ^2^					
≥2 partners for casual sex per month	3.275	26.43	8.50	82.24	<0.001
Disclosing serologic status on dating apps	0.757	2.13	1.91	2.36	<0.001
Being in a polyamorous relationship	0.587	1.80	1.50	2.15	<0.001
Being single	0.319	1.37	1.18	1.59	<0.001
Having sex with unknown partners	0.223	1.25	1.11	1.40	<0.001
Level of education	0.223	1.25	1.12	1.40	<0.001
*Engaging in challenging sexual practices	0.203	1.22	1.10	1.35	<0.001
Group sex	−0.194	0.82	0.73	0.92	0.001
*Immigrants multivariate analysis* ^3^					
≥2 partners for casual sex per month	2.185	8.89	2.96	26.66	<0.001
Disclosing serologic status on dating apps	0.816	2.26	1.93	2.65	<0.001
Being single	0.334	1.40	1.15	1.68	0.001
^*^Engaging in challenging sexual practices	0.262	1.30	1.12	1.50	<0.001

CI95%, confidence interval; aPR, adjusted prevalence ratio/Best Akaike and likelihood criteria were evaluated. Model 1 and 3. Adjusted for routine HIV testing. Model 2. Adjusted for routine testing and known HIV status.

1 Omnibus test (*p* < 0.001)/ROC curve: 0.929 (0.922–0.936; *p* < 0.001); AIC: 3,829.29; LLR: −1,902.65; Deviance: 1,573.29.

2 Omnibus test (*p* < 0.001)/ROC curve: 0.939 (0.932–0.947); *p* < 0.001; AIC: 2,759.33; LLR: −1,367.66; Deviance: 1,159.33.

3 Omnibus test (*p* < 0.001)/ROC curve: 0.927 (0.911–0.943); *p* < 0.001; AIC: 1,022.37; LLR: −503.19; Deviance: 350.37.

* Engaging in at least one of challenging sexual practices: cruising, chemsex, fisting, and/or double penetration.

Some factors were uniquely associated with Brazilian MSM residents in higher prevalence for PrEP use when compared to immigrants living in Portugal: being in a polyamorous relationship, having sexual relations with unknown partners, and high education level. Practitioners of group sex have a 10% lower prevalence of using PrEP. On the other hand, Brazilian immigrants living in Portugal have four characteristics that indicate higher PrEP use, such as: having more than two sexual partners in 30 days, declaring their serological status on dating apps, being single, and being adepts of challenging sexual practices. It is possible to highlight that the group of factors that impact higher PrEP use in immigrants is also contained among those that impact Brazilian MSM residents (Table 3).

## 4. Discussion

This study aims to shed light on the use of PrEP among both native and immigrant Brazilian MSM, focusing on prevention options and strategies within the context of global health. Our results indicate a low prevalence of PrEP use among Brazilians, both nationally and among Brazilian immigrants living in Portugal (6, 21–23). Interestingly, we also observed a convergence of factors associated with high PrEP use among Brazilian residents and immigrants. These findings have significant implications for the development of public health policy in global health, particularly in ensuring continued access to PrEP for individuals who have migrated from Brazil to other countries, such as Portugal. Our research highlights the importance of sustained access to PrEP for these individuals, supports ongoing PrEP use, and advocates for the development and implementation of policies that prioritize the health and well-being of this population. Emphasizing the necessity of such policies is crucial.

The international community has committed to the Sustainable Development Goal (SDG) of ending the HIV/AIDS epidemic by 2030, with PrEP being an important component of the existing HIV prevention package for those engaging in higher-risk sexual behaviors. In 2015, the WHO recommended offering PrEP as an additional prevention option for individuals at substantial risk of HIV infection. It is estimated that 3 million people worldwide should use PrEP annually to achieve the SDGs, with a focus on key populations and those at higher risk in high-prevalence settings.

The implementation of PrEP in Europe has been based on WHO recommendations and is rapidly changing with the availability and increased access to generic PrEP (emtricitabine and tenofovir) through national health systems on the continent. However, immigrants still face difficulties, especially those who are undocumented or irregular, as they do not have their rights protected by laws and agreements between the two countries. This can limit their access to public health services. As of 2021, only 15 out of 50 European countries reported providing PrEP for irregular migrants, while four other countries reported that PrEP was only available for irregular migrants through private clinics or at a cost. Despite Portugal being among the countries that provide free PrEP, this information may not be clear and readily available to the population, resulting in a low prevalence of use among the immigrant Brazilian participants studied.

On the other hand, for Brazilians in the country, although Brazil invests heavily in PrEP, there are still challenges in obtaining and maintaining its use. More vulnerable populations, such as MSM, trans people, and sex workers, face additional barriers to accessing PrEP, including stigma and discrimination, lack of information about PrEP availability, and difficulties in accessing health services. PrEP is only distributed in certain larger cities, limiting access for individuals living in more rural and remote areas or those without access to specialized health services. Additionally, the lack of clear and accessible information on how to obtain PrEP through the public health system, as well as delays and bureaucratic processes, discourage individuals from seeking and using PrEP.

In this study, we included Brazilian residents in the country and Brazilian immigrants living in Portugal to compare the factors associated with PrEP use between the different subgroups. It is important to highlight that according to multivariate models, four converging factors increased the prevalence of PrEP use among Brazilians regardless of migration status: having 2 or more casual sexual partners per month, engaging in challenging sexual practices, disclosing serostatus on apps, and being single.

The first of these factors refers to a high number of unknown sexual partners, which, when combined with the “being single” factor, promotes multiple partnerships. Having multiple sexual partners or not undergoing HIV testing are increased risk factors for HIV infection, as they raise the likelihood of coming into contact with someone who is HIV-positive. This is particularly concerning when one is unaware of their partners’ serostatus (24, 25). Research suggests that migration can be a risk factor for engaging in risky sexual behaviors, including an increase in the number of sexual partners (8). This may be attributed to various factors, such as exposure to new environments and cultures, difficulty in establishing stable relationships due to being away from family and friends, social pressure to conform to new behavioral patterns, among others (26, 27).

On the other hand, the finding that “declaring serostatus on apps” can increase the prevalence of PrEP use may indicate that Brazilian MSM, regardless of their immigrant or native status, are utilizing alternative biomedical prevention methods for HIV through disclosing their serostatus on dating apps. Disclosing HIV serostatus on dating app profiles can facilitate dialog and negotiation regarding the adoption of preventive measures even before engaging in sexual activity itself, respecting the desires and possibilities of all parties involved (28).

In some apps, it is possible to indicate HIV serostatus in the profile section. Publicly stating that one is on PrEP in their dating app profile can help increase knowledge about and adherence to PrEP among this population. Publicly declaring one’s PrEP usage can contribute to reducing stigma associated with medication or seropositivity, normalizing discussions about HIV prevention (26). Furthermore, publicly stating that one is on PrEP can raise awareness about the importance and popularity of PrEP and help dispel misinformation about the medication (6). Finally, publicly indicating PrEP usage can help connect app users who are interested in engaging with others who are on PrEP, maximizing protection against HIV (15). Additionally, an app user who is on PrEP can share their experience with the medication and encourage other users to consider it as an option for HIV prevention. Brazilians, especially those who immigrate, have limited knowledge about PEP and PrEP, so providing avenues to disseminate this knowledge among peers is crucial.

A common third factor was the engagement in challenging sexual practices, such as double penetration, fisting or footing, and cruising. The concept of challenging sexual practices for MSM may vary depending on the culture and subculture they are part of. However, it should be understood as a combination of practices that can impact HIV exposure and facilitate the adoption of less effective prevention methods (7). Combining sexual practices like fisting, footing, and double penetration, for instance, may increase the risk of injuries, leading to the transmission of STIs (6). Given the potential for injuries and exposure to bodily fluids, it is essential to provide access to PrEP for individuals who engage in these activities and monitor their adherence.

The adoption of challenging sexual practices can render both immigrant and native MSM vulnerable. However, vulnerability may be more pronounced for immigrant MSM due to the accumulation of multiple social stressors they face, such as stigma associated with sexual orientation and immigration, barriers to accessing health and prevention services, and challenges in adapting to new cultural environments (29). Immigrant MSM may also encounter cultural and linguistic barriers when accessing sexual health information and services, which can increase their vulnerability to STI exposure and reduce their self-efficacy in adopting preventive measures like PrEP (30).

Regarding the factors associated with PrEP use among native Brazilians, three distinct factors were identified: being in a polyamorous relationship, engaging in sexual activity with unknown casual partners, and higher education.

Several studies have established a positive correlation between education level and PrEP use. For instance, a study conducted in the United States in 2019 that assessed PrEP adherence in a representative sample of MSM found higher adherence among individuals with higher education levels (e.g., bachelor’s or post-graduate degrees) (31). Another study with MSM in the United States indicated that education level was a strong predictor of PrEP knowledge and current use, with men having higher education levels being more likely to be aware of and use PrEP compared to those with lower education levels (32).

These findings are further supported by a study conducted in the city of São Paulo, Brazil, in 2020, which suggested a positive association between education and PrEP adherence among MSM (33). Possible explanations for this association include higher health literacy, improved access to health information, enhanced health decision-making skills, and greater access to quality health services and medical care.

The study demonstrated that being in a polyamorous relationship and engaging in sexual activity with unknown casual partners are indicative of PrEP use. However, having more frequent relationships with multiple partners may expose these MSM to various situations of HIV/AIDS exposure. In one study, researchers evaluated PrEP utilization in a sample of gay and bisexual men in polyamorous relationships in the United States, revealing that 28% of them had previously used PrEP, and among those who had not used it, 56% expressed a high interest in utilizing PrEP (34).

Individuals in polyamorous relationships may be more conscious of the higher likelihood of encountering unknown sexual partners, especially if the relationship is non-monogamous or if there is a policy of not discussing a partner’s HIV serostatus before engaging in sexual activity, which increases the chances of exposure and emphasizes the recommendation for PrEP use (35, 36).

In our study, we found that only one factor was associated with a lower prevalence of PrEP use among Brazilians residing in the country: the practice of group sex. Although this result may seem unusual, it can probably be related to low risk perception among certain subgroups involved in high-risk sexual practices for HIV exposure. An example of this is drug-associated or combined sex (chemsex) where group sex is often associated and can interfere with the consistent and correct use of PrEP (37–39). This finding reinforces and highlights the need for targeted interventions and education about the benefits of PrEP in these specific contexts (40–42).

To effectively expand the implementation of PrEP and bridge the gap between current levels and target goals, it is crucial to comprehensively understand the factors associated with PrEP utilization among Brazilians, regardless of their migration status (whether they are migrants or residents in the country). The results of our study highlight the convergence of these factors and provide valuable insights for the development of personalized strategies that capitalize on the specific characteristics of this population. By doing so, we can enhance behavior identification and risk factor assessment, leveraging these insights to increase PrEP uptake and inform the direction of public policies aimed at prioritizing PrEP engagement among key populations, particularly MSM (43, 44).

Firstly, public policies should be directed toward increasing the availability and accessibility of PrEP, especially in Brazil, in order to meet the demand, particularly among priority groups such as MSM. We believe that PrEP use among this sample of MSM is low and that it should be increased. This can be achieved by expanding the distribution of PrEP beyond hospitals and involving community organizations in the dispensing and planning of PrEP, not just in case screening. Promoting collaboration between healthcare professionals and community organizations supporting MSM or immigrants can provide unique and culturally sensitive resources (6, 43).

Our data showed a convergence of factors among Brazilians, regardless of migration status, mostly related to partnerships and sexual practices. Therefore, approaches should be able to identify these characteristics in participants, improving the ability to identify potential PrEP users and sensitizing them to the need for PrEP. Brazilian MSM engaging in the sexual practices identified in this study may be more likely to seek PrEP if they understand their individual risk of HIV and how PrEP can help reduce that risk. This can be facilitated through individual risk assessments and counseling by healthcare professionals (43).

Our study highlighted the importance of strengthening strategies to improve adherence to PrEP use in key populations, considering the global health perspective and population movements, especially for populations migrating between countries. However, Brazil and Portugal may face several challenges in creating and implementing public policies in this context, including issues such as illegal immigration, legal and regulatory barriers, funding constraints, planning difficulties, and limited data on factors influencing acceptance and adherence to PrEP among immigrant populations.

One strategy that can be implemented is to utilize airport spaces as locations for the dissemination of available services within each country’s healthcare networks, such as offering care and dispensing supplies and medications. Additionally, communication and health education strategies for HIV prevention and treatment in the context of migration should be expanded. Therefore, it is necessary to encourage and promote courses that provide education and facilitate access to rights, such as the course offered by the International Organization for Migration and the Pan American Health Organization, with a focus on sexual health and migration from the perspective of vulnerabilities, diversity, rights, sexuality, reproduction, discrimination, and sexually transmitted infections (45). Initiatives like these can be replicated in other countries to reach the migrant population and engage prevention agents.

Our research has certain limitations that should be taken into account. Firstly, the study design was observational, which means that it is not possible to establish a cause-effect relationship between the analyzed factors, only associations. Secondly, our data were collected online, relying on self-reported information, which makes it difficult to verify its accuracy and truthfulness. Thirdly, the use of electronic forms and the requirement for reading and instructions to answer the questions limited the sample to MSM with a certain level of education and purchasing power, as they needed access to a smartphone/computer and the internet. This may have introduced a significant selection bias in this sample of immigrants.

It is also important to consider that the research was conducted during the COVID-19 pandemic, and the associated isolation measures may have had an impact on the results. Health facilities were either closed or overcrowded, which may have affected the participants’ willingness and ability to seek and access PrEP. Lastly, the use of snowball sampling should be acknowledged as a limitation since it does not allow for generalizing the results to the overall population of immigrant MSM.

On the strengths of this study, it is the first to comparatively investigate social, demographic, partnership, and sexual practices and behaviors among a sample of Brazilians living in the country and immigrants. Our findings provide a basis for discussing public policies that enable the continuation of PrEP use for individuals who have migrated from Brazil to Portugal, such as establishing a system that recognizes and validates PrEP prescriptions issued in Brazil. This could involve the creation of agreements or protocols between the healthcare systems of the two countries, allowing immigrants to maintain their PrEP use in Portugal.

Furthermore, such a policy would demonstrate a commitment to the health and well-being of immigrants, as suggested by SDGs 3 and 10, recognizing the importance of continued HIV treatment and prevention regardless of country of origin. This would strengthen the global response to HIV and promote equity in access to healthcare for migrant populations.

## 5. Conclusion

The attention to migrant men using PrEP should be directed on a larger scale, considering that they are a population that is already sensitive to adherence to HIV prevention care technology, more so than others. However, they may face barriers in adherence and maintenance of the treatment experienced in the countries, which constitutes an issue of international health concern.

Our findings indicate that, regardless of migration status, in our sample of participants, having 2 or more casual sexual partners per month; engaging in challenging sexual practices; declaring serological status on apps, and being single increase the likelihood of PrEP use among Brazilians. Individually, being in a polyamorous relationship, having sexual relations with unknown partners, and having a high education level increased the likelihood of PrEP use among Brazilians living in Brazil. These factors should be taken into account in the implementation of strategies to strengthen PrEP adherence in Brazil and in the creation of international programs that allow its use among populations migrating between these two countries.

## Data availability statement

The raw data supporting the conclusions of this article will be made available by the authors upon reasonable request to the corresponding author.

## Ethics statement

The studies involving humans were approved by Research Ethics Committee of the IHMT of Universidade Nova de Lisboa (Protocol nº 12.19/2020). The studies were conducted in accordance with the local legislation and institutional requirements. The participants provided their written informed consent to participate in this study.

## Author contributions

ÁS, SL, CR, AS, EC, LO, JN, IM, and IF: conceptualization, funding acquisition, methodology, software, validation, formal analysis, investigation, resources, data curation, writing—original draft preparation, and review and editing. ÁS, SL, and CR: software, validation, formal analysis, data curation, writing—original draft preparation, and review and editing. All authors contributed to the article and approved the submitted version.

## Funding

This work was supported by National Research Council–CNPq Process: 159908/2019-1. The funders had no role in the study design; in data collection, analyses, or interpretation; in writing of the manuscript; or in the decision to publish the results.

## Conflict of interest

The authors declare that the research was conducted in the absence of any commercial or financial relationships that could be construed as a potential conflict of interest.

## Publisher’s note

All claims expressed in this article are solely those of the authors and do not necessarily represent those of their affiliated organizations, or those of the publisher, the editors and the reviewers. Any product that may be evaluated in this article, or claim that may be made by its manufacturer, is not guaranteed or endorsed by the publisher.
